# The seminal vesicle is a juvenile hormone-responsive tissue in adult male *Drosophila melanogaster*

**DOI:** 10.1098/rsob.240315

**Published:** 2024-12-18

**Authors:** Yoshitomo Kurogi, Yosuke Mizuno, Ryosuke Hayashi, Krystal Goyins, Naoki Okamoto, Lacy Barton, Ryusuke Niwa

**Affiliations:** ^1^ Graduate School of Science and Technology, University of Tsukuba, Ibaraki 305-8577, Japan; ^2^ Degree Programs in Life and Earth Sciences, Graduate School of Science and Technology, University of Tsukuba, Tsukuba, Japan; ^3^ Department of Neuroscience, Developmental and Regenerative Biology, University of Texas at San Antonio, One UTSA Circle, San Antonio, TX 78249, USA; ^4^ Life Science Center for Survival Dynamics, Tsukuba Advanced Research Alliance (TARA), University of Tsukuba, Ibaraki 305-8577, Japan

**Keywords:** *Drosophila melanogaster*, juvenile hormone, juvenile hormone response element-GFP, lactate dehydrogenase, seminal vesicle

## Abstract

Juvenile hormone (JH) is one of the most essential hormones controlling insect metamorphosis and physiology. While it is well known that JH affects many tissues throughout the insect life cycle, the difference in JH responsiveness and the repertoire of JH-inducible genes among different tissues has not been fully investigated. In this study, we monitored JH responsiveness *in vivo* using transgenic *Drosophila melanogaster* flies carrying a *JH response element-GFP* (*JHRE-GFP*) construct. Our data highlight the high responsiveness of the epithelial cells within the seminal vesicle, a component of the male reproductive tract, to JH. Specifically, we observe an elevation in the JHRE-GFP signal within the seminal vesicle epithelium upon JH analogue administration, while suppression occurs upon knockdown of a gene encoding the intracellular JH receptor, *germ cell-expressed*. Starting from published transcriptomic and proteomics datasets, we next identified *Lactate dehydrogenase* as a JH-response gene expressed in the seminal vesicle epithelium, suggesting insect seminal vesicles undergo metabolic regulation by JH. Together, this study sheds new light on the biology of the insect reproductive regulatory system.

## 1. Introduction

Juvenile hormone (JH) was initially discovered in the 1930s as an insect metamorphosis inhibition factor [1–4]. JH is synthesized in the *corpora allata* (CA) and regulates many aspects of insect physiology throughout the life cycle [5–8]. JH signalling is mediated through intracellular JH receptors, Methoprene-tolerant (Met) and its orthologues, which belong to the basic helix-loop-helix (bHLH)-Per-Arnt-Sim (PAS) family of transcriptional factors [9–12]. Met and its orthologous transcription factors bind to JH with high affinity [10,13]. Upon JH binding, these intracellular receptors associate with specific JH response elements (JHREs), containing a C-box sequence (CACGCG, an E-box-like motif) or a canonical E-box sequence (CACGTG) [13], followed by the transcriptional induction of target genes, such as *Krüppel-homolog 1* (*Kr-h1*) [13–18].

In the last decade, the fruit fly *Drosophila melanogaster* has contributed to elucidating molecular mechanisms of JH-responsiveness [19]. Two intracellular JH receptors have been identified in *D. melanogaster*, known as Met and Germ cell-expressed (Gce). Single loss-of-function of either *Met* and *gce* is adult viable, while double mutants of *Met* and *gce* result in developmental arrest during pupation, like CA-ablated flies [9,20], suggesting that Met and Gce act redundantly to regulate JH-responsive gene expression [10]. A recent study using *GAL4*- and *LexA*-based reporters showed that *Met* and *gce* are both broadly expressed in many, but not all, tissues throughout *D. melanogaster* development [21], suggesting many tissues have the potential to transcriptionally respond to JH. Yet, whether all tissues that express JH receptors have active JH transcriptional signalling is unknown.

To approach this problem, we conducted a study using a *D. melanogaster* strain carrying a *JH response element-GFP* (*JHRE-GFP*) [22]. The *JHRE-GFP* construct contains eight tandem copies of a JHRE, originally identified from the *early trypsin* gene of *Aedes aegypti* [16,17,23]. It has also been confirmed that *JHRE* is responsive to JH analogues (JHAs) in *D. melanogaster* S2 cultured cells [10]. In addition, a recent study has shown that GFP signals of *JHRE-GFP* transgenic flies can monitor JH-responsiveness in *D. melanogaster* embryos [22].

In this study, we show that JHRE-GFP signal is found in epithelial cells of the adult seminal vesicle, which is a part of the male reproductive tract in *D. melanogaster*. The JHRE-GFP signal in the seminal vesicle epithelium is elevated upon administration of the JHA, methoprene and conversely suppressed in animals depleted of *gce* by RNAi. We also show that JHRE-GFP in the seminal vesicle epithelium is elevated after mating, consistent with a previous hypothesis that mating elevates JH titer in male adults [24,25]. Furthermore, we identified *Lactate dehydrogenase* (*Ldh*) as a JH-response gene expressed in the seminal vesicle epithelium. Our study demonstrates the seminal vesicle as a novel JH-responsive tissue in *D. melanogaster*.

## 2. Results

### 2.1. The seminal vesicle in male *D. melanogaster* is a JH-responsive tissue

In previous studies, while the functions of JH during development and its effects on the reproductive system of adult females have been extensively studied [1–8,19], its functions in adult males have received less investigation. Therefore, we investigated which cells/tissues are responsive to JH in the adult males using *JHRE-GFP* transgenic flies. Whereas *JHRE-GFP* strain has been used for monitoring JH-responsive cells during embryogenesis [22], it has not been used for adult males. Therefore, we first examined JHRE-GFP fluorescence signals in whole male adult bodies. We used two strains in this study, namely *JHRE^Wild-type (WT)^-GFP* males with *JHRE^Mutated (Mut)^-GFP* males [22]. *JHRE^WT^-GFP* strain carries a wild-type JHRE, while *JHRE^Mut^-GFP* strain carries a mutated JHRE in which Met and Gce binding sites are disrupted [10,22]. We observed strong GFP signals in the scattered hemocytes and some tissues in the abdominal region of *JHRE^WT^-GFP*, but not *JHRE^Mut^-GFP* flies (electronic supplementary material, figure S1*a*). We also orally administrated methoprene to these animals and found that the GFP signals in the abdomen were particularly elevated in *JHRE^WT^-GFP*, but not *JHRE^Mut^-GFP* flies (electronic supplementary material, figure S1*a*, arrowhead). Based on the data, we further anatomically characterized where *JHRE^WT^-GFP* was expressed in abdominal tissues.

**Figure 1.**
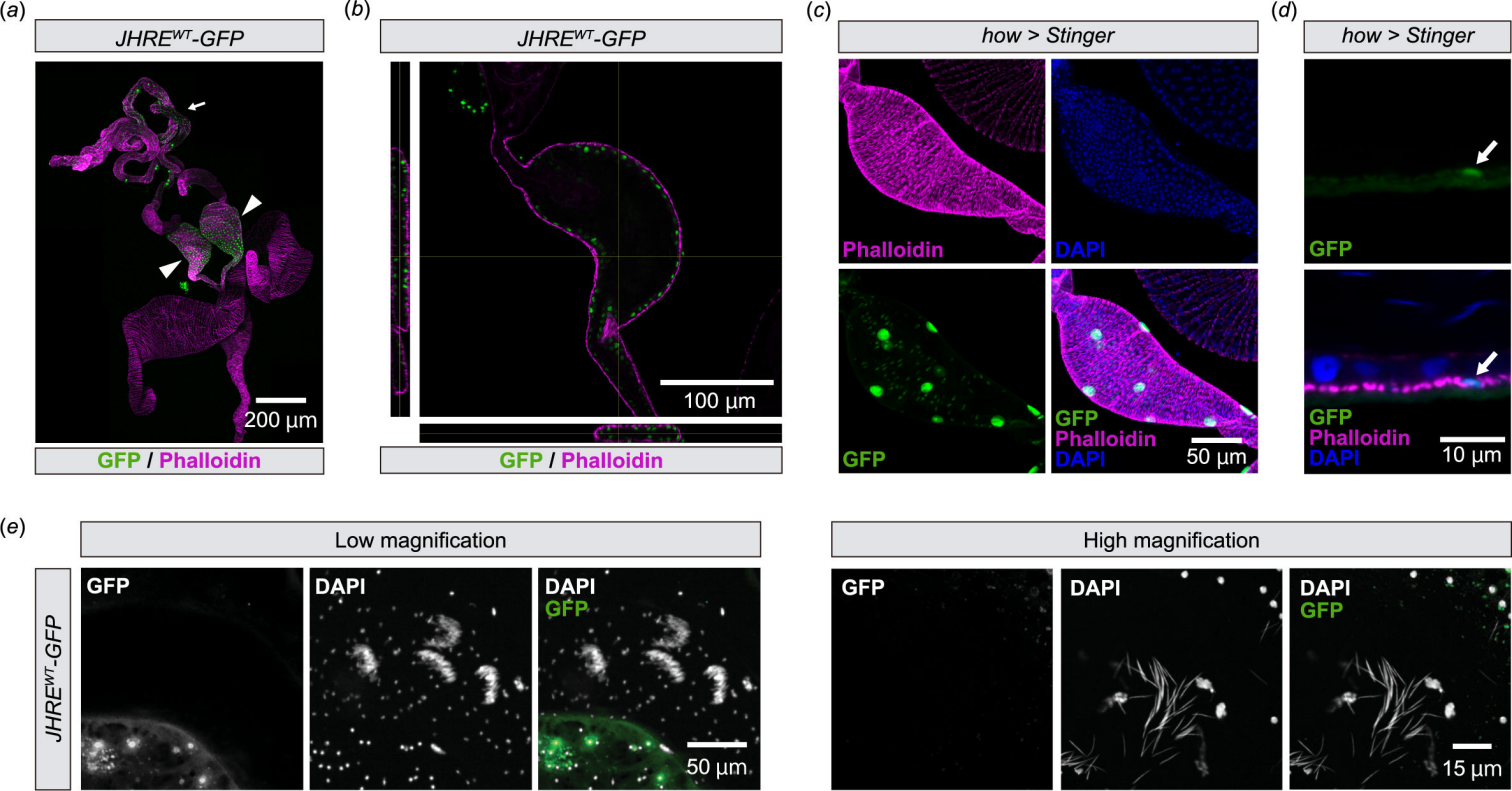
*JHRE-GFP* is expressed in seminal vesicle epithelial cells (*a,b*) Immunostaining with anti-GFP (green) and phalloidin (magenta) of *JHRE^WT^-GFP* adult male. (*a*) Image of the male reproductive tract. The arrowhead and the arrow indicate the seminal vesicles and the testis, respectively. (*b*) Cross-section image of the seminal vesicle. Left and bottom images indicate horizontal and vertical cross-sectional views, respectively. (*c,d*) Transgenic visualization of muscles by nuclear GFP (Stinger) driven by *how-GAL4*. Samples were immunostained with anti-GFP antibody (green), phalloidin (magenta) and DAPI (blue). Samples were derived from virgin males 2 days after eclosion. (*c*) Image of the seminal vesicle. (*d*) Magnified view of the seminal vesicle epithelial cells. The arrow indicates a cell with GFP signal. (*e*) Immunostaining of sperm with anti-GFP (green) and DAPI (white) in *JHRE^WT^-GFP* adult male.

Dissection of *JHRE^WT^-GFP* male abdomens revealed that the JHRE-GFP signal was present in a part of the male reproductive tract, including the testes and seminal vesicles (figure 1*a*), which is known to store sperm produced in the testis [26]. As the seminal vesicle showed the most remarkable JHRE-GFP signal in the male reproductive tract, we decided to focus on this tissue for the rest of this study. Within the seminal vesicles, JHRE-GFP was active in cells located on the lumen side compared with the muscle layer surrounding the seminal vesicle labelled with fluorescence-conjugated phalloidin (figure 1*b*). We assume that these luminal side cells were not muscle cells but epithelial cells, as *GFP* driven by the muscle driver *how-GAL4* [27] was expressed in fewer cells than JHRE-GFP-positive cells in the seminal vesicles (figure 1*c*) and embedded in the phalloidin-positive muscle layer (figure 1*d*). In addition, we found that the JHRE-GFP signal was not observed in extracted sperm (figure 1*e*). Together, these results suggest that *JHRE-GFP* is expressed in the seminal vesicle epithelial cells.

We also examined whether these cells were labelled with another JH reporter strain, *JH response region* (*JHRR*)*-LacZ. JHRR-LacZ* is a *LacZ* reporter fused with the JHRR of the *D. melanogaster Kr-h1* promoter, which is responsive to JH via Met and Gce [14]. We found that *JHRR-LacZ* was also expressed in the seminal vesicle cells, some cells in the testes just anterior to the seminal vesicle and some secondary cells of the male accessory gland (electronic supplementary material, figure S1*b*). Similar to *JHRE-GFP*, *JHRR-LacZ* also labelled the epithelial cells of the seminal vesicles (electronic supplementary material, figure S1*c*,*d*).

**Figure 2.**
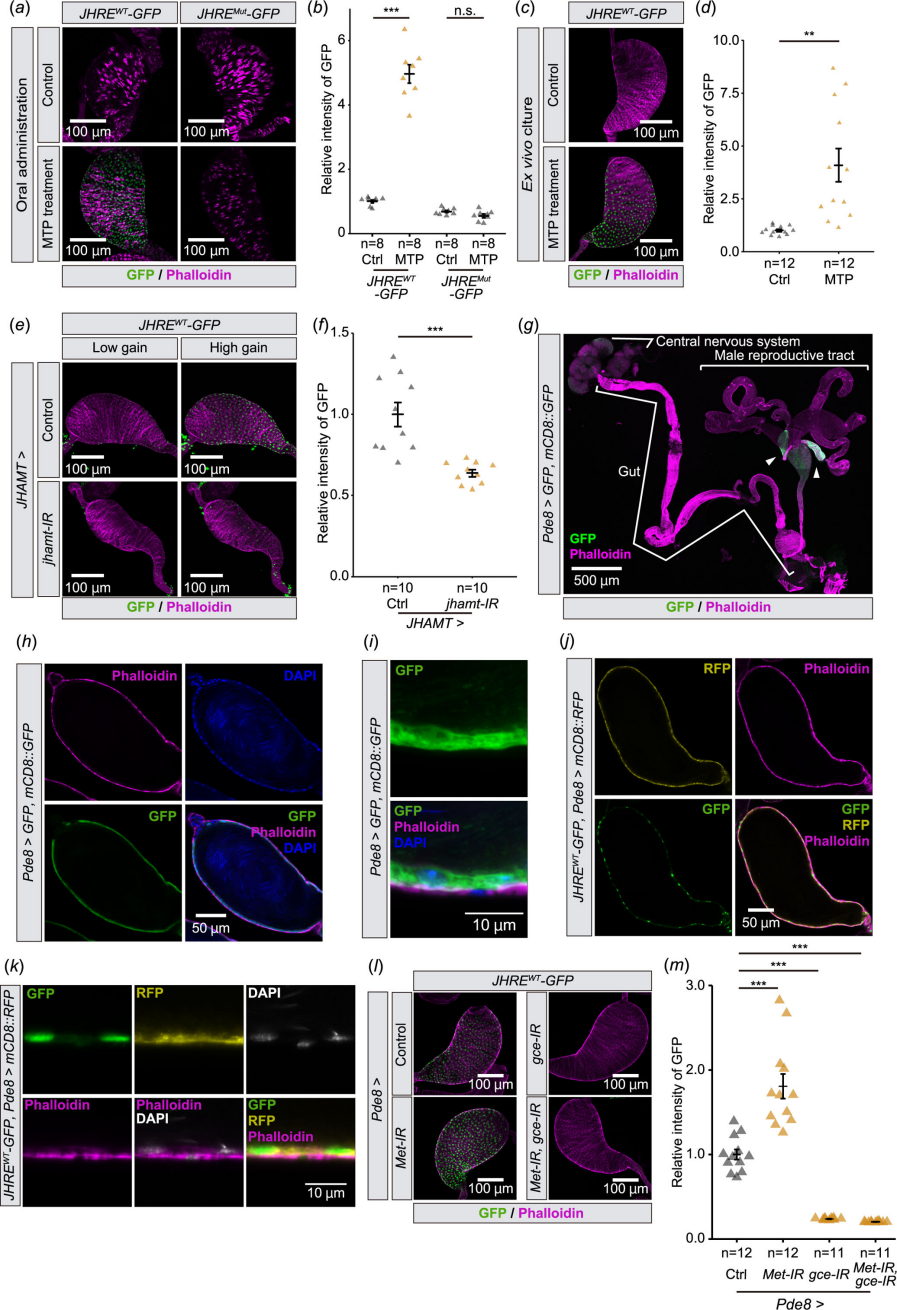
JHRE-GFP signal in the seminal vesicle changes depending on JH signalling. (*a,b*) JHRE-GFP signal in the seminal vesicle of *JHRE^WT^-GFP* and *JHRE^Mut^-GFP* males 7 days after eclosion with or without oral administration of methoprene (MTP). (*a*) Representative images of the seminal vesicles immunostained with GFP and phalloidin (F-actin) shown in green and magenta, respectively. (*b*) Quantification of JHRE-GFP signals in the seminal vesicles of control (Ctrl) and MTP-administered males. (*c,d*) JHRE-GFP signals in the seminal vesicle of *JHRE^WT^-GFP* males 4 days after eclosion. Male reproductive tracts without male accessory glands are cultured *ex vivo* with or without MTP. (*c*) Representative images of the seminal vesicles immunostained for GFP (green) and phalloidin (magenta). (*d*) Quantification of JHRE-GFP signal in the seminal vesicles. (*e,f*) JHRE-GFP signal in the seminal vesicle of control and *JHAMT-GAL4*-driven *jhamt* RNAi males 4 days after eclosion. Control RNAi was achieved with a VDRC KK control line. GFP and phalloidin (F-actin) signals are shown in green and magenta, respectively. (*e*) Representative images of the seminal vesicles. ‘Low gain’ GFP signals were captured with the same gain as shown in (*c*). ‘High gain’ GFP signals were captured with 1.23-fold gain setting compared with ‘Low gain’ (800vs650). GFP and phalloidin (F-actin) signals are shown in green and magenta, respectively. (*f*) Quantification of JHRE-GFP signal in the seminal vesicle. (*g,i*) Immunostaining with anti-GFP (Green), phalloidin (Magenta), and DAPI (Blue) of males carrying *Pde8-GAL4* along with both *UAS-GFP* and *UAS-mCD8::GFP* 4 days after eclosion. (*g*) Image of the central nervous system, gut and male reproductive tract. Arrowheads indicate the seminal vesicles. (*h*) Cross-section image of the seminal vesicle. (*i*) Magnified view of the seminal vesicle epithelial cells. (*j,k*) Immunostaining with anti-GFP (Green), anti-RFP (Magenta), phalloidin (Blue) and DAPI (White) of *JHRE-GFP^WT^*, *Pde8-GAL4 UAS-mCD8::RFP* males 4 days after eclosion. (*j*) Cross-section image of the seminal vesicle. (*k*) Magnified view of the seminal vesicle epithelial cells. (*l,m*) JHRE-GFP signal in the seminal vesicle of control males and *Pde8-GAL4*-driven *Met* and/or *gce* RNAi males 7 days after eclosion. Note that this experiment was conducted with food supplemented with MTP, as the MTP administration allowed us to see more drastic difference in JHRE-GFP signals between control and RNAi. Control flies were obtained by crossing *w^1118^* with *Pde8-GAL4* driver. (*l*) Representative images of the seminal vesicles. (*m*) Quantification of JHRE-GFP signal in the seminal vesicle. Values in *b*,*d*,*f* and *m* are presented as mean ± s.e. Statistical analysis: Student’s *t*‐test for *b*,*d*,*f*. Wilcoxon rank sum exact test with Bonferroni’s correction for *m*.**p < 0.01 ***p < 0.001. n.s. not significant.

We next examined whether seminal vesicle cells respond to the JH signalling. We found that the oral administration of methoprene increased JHRE-GFP signal in the seminal vesicles of virgin males carrying the *JHRE^WT^-GFP*, but not *JHRE^Mut^-GFP*, transgene (figure 2*a*,*b*). In addition, JHRE-GFP signal was elevated in *ex vivo* cultured seminal vesicles 16 h after incubation with methoprene (figure 2*c*,*d*), suggesting that the seminal vesicle itself responds to JH. Conversely, when JH biosynthesis was blocked by knocking down *JH acid O-methyltransferase* (*jhamt*), a rate-limiting enzyme for JH biosynthesis in the CA [28,29], JHRE-GFP signal in the seminal vesicle decreased (figure 2*e*,*f*). These results suggest that the seminal vesicle epithelial cells respond to changes in circulating JH.

We also examined whether *JHRR-LacZ* expressed in seminal vesicles is upregulated by methoprene feeding. However, anti-LacZ immunostaining signal in the seminal vesicle was not increased by methoprene feeding in *JHRR-LacZ* flies (electronic supplementary material, figure S1*e*,*f*). Since the JH dependence of *JHRR-LacZ* expression in seminal vesicles was unclear, we used *JHRE-GFP* as a JH-responsive marker in subsequent analyses.

### 2.2. JH signalling in the seminal vesicle requires Met and Gce

We next confirmed that *JHRE-GFP* expression in the seminal vesicle was mediated by intracellular JH receptors, Met and Gce [13–18]. However, since *Met* and *gce* double mutant flies die during the larval-pupal transition [9], we conducted transgenic RNAi to knockdown *Met* and *gce* with a GAL4 driver that labels the seminal vesicle epithelial cells. After our GAL4 driver screen (see §4 for details), we found that *Pde8-GAL4* driver drives gene expression in the seminal vesicles (figure 2*g*). Our further detailed analysis confirmed that *Pde8-GAL4* labels the seminal vesicle epithelial cells (figure 2*h*,*i*). In addition, cells labelled with JHRE^WT^-GFP colocalized with *Pde8*>*mCD8::RFP* seminal vesicle epithelial cells (figure 2*j*,*k*). Using this GAL4 driver, we found that JHRE-GFP signal in the seminal vesicle epithelial cells was decreased by *Met* and *gce* double knockdown (figure 2*l*,*m*). Furthermore, a reduction in JHRE-GFP signalling was also seen in *gce* knockdown flies, but not in *Met* knockdown flies (figure 2*l*,*m*). These results suggest that JH is received mainly by Gce in the seminal vesicle epithelial cells.

### 2.3. Mating activates JH signalling in the seminal vesicle

**Figure 3.**
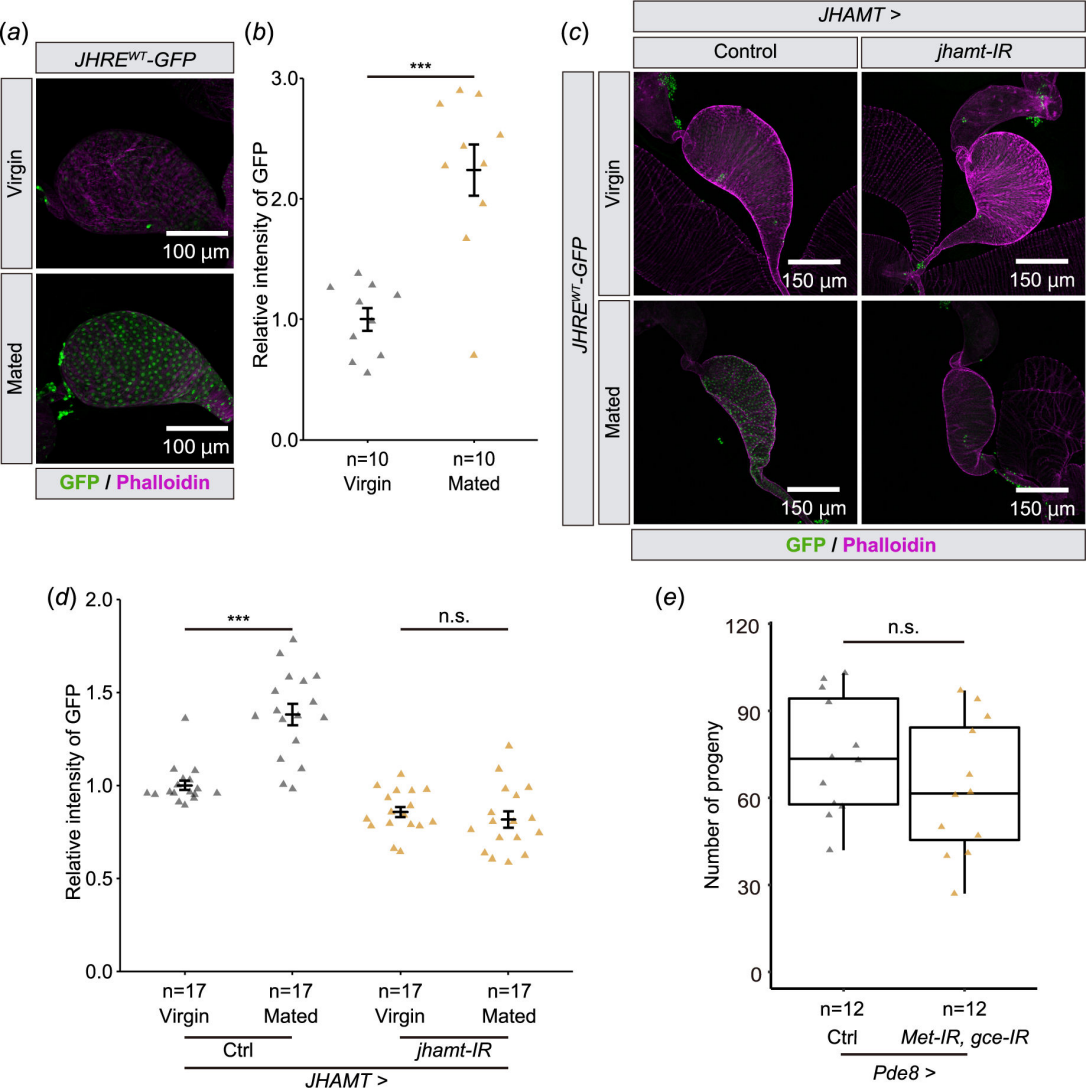
JHRE-GFP signal in the seminal vesicle is increased after mating. Samples were derived from males 6 days after eclosion. In all photos, GFP and phalloidin (F-actin) signals are shown in green and magenta, respectively. (*a,b*) JHRE-GFP signals in the seminal vesicle of virgin or mated males. (*a*) Representative images of the seminal vesicles. (*b*) Quantification of JHRE-GFP signals in the seminal vesicles. (*c,d*) JHRE-GFP signals in the seminal vesicles of control and *JHAMT-GAL4*-driven *jhamt* RNAi males with or without mating. Control RNAi was achieved with VDRC KK control line noted in the Methods section. (*c*) Representative images of the seminal vesicles. (*d*) Quantification of JHRE-GFP signals in the seminal vesicles. (*e*) Number of progeny from control and *Pde8-GAL4*-driven *Met* and *gce* RNAi males after mating. Control was *Pde-GAL4* males crossed to *w^1118^* females. Control flies were obtained by crossing *w^1118^* with *Pde8-GAL4* driver. Values in *b* and *d* are presented as mean ± s.e.. Statistical analysis: Student’s *t*‐test for *b*. Tukey–Kramer test for *d*. Wilcoxon rank sum exact test for *e*. *p < 0.05, ***p < 0.001. n.s.: not significant.

Next, we tested whether *JHRE-GFP* expression in the seminal vesicle is responsive to natural processes reported to impact JH signalling. In *D. melanogaster* males, JH signalling may increase in a mating-dependent manner [24,25]. These previous observations motivated us to compare JHRE-GFP signals in the seminal vesicle between virgin and mated males. We found that JHRE-GFP signal in the seminal vesicle epithelial cells increased in mated males as compared with virgin males (figure 3*a*,*b*). In addition, the increase of the JHRE-GFP signal upon mating was reduced by *jhamt* RNAi using the *JHAMT-GAL4* driver (figure 3*c*,*d*). These results suggest that JH signalling in the seminal vesicle epithelium is responsive to mating. These results raise the possibility that JH signalling in the seminal vesicle influences fertility after mating. However, a double knockdown of *Met* and *gce* did not impact on the number of progeny (figure 3*e*). This result indicates that JH signalling in the seminal vesicle does not play a major role in fertility.

### 2.4. JH induces expression of Ldh in the seminal vesicle

**Figure 4.**
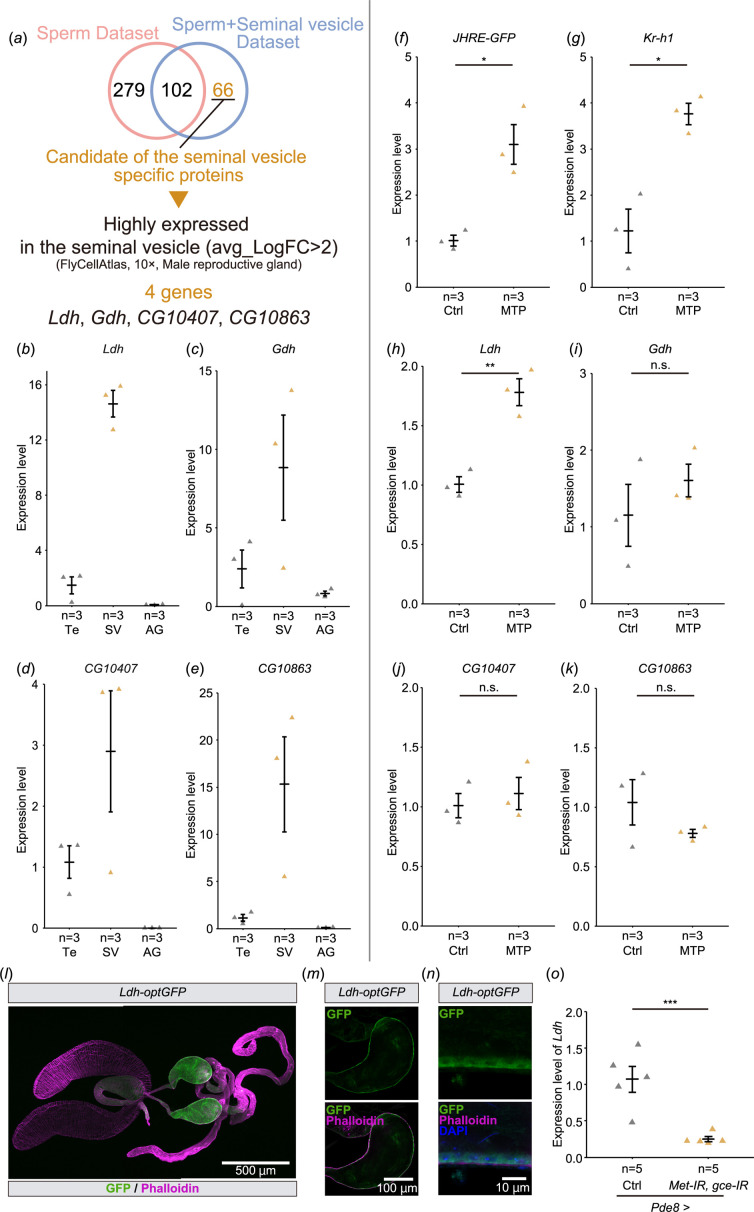
Screening of genes highly expressed in the seminal vesicle. (*a*) A flowchart to identify candidate genes that are highly and predominantly expressed in the seminal vesicles. See §§2 and 4§ for details. (*b–e*) Reverse transcription-quantitative PCR (RT-qPCR of the candidate genes in *JHRE^WT^-GFP* males. mRNA levels were compared among the testes (Te), seminal vesicles (SV), and male accessory glands (AG). Each dot represents the levels of mRNA derived from 8 virgin males 6 days after eclosion. (*b*) *Ldh*. (*c*) *Gdh*. (*d*) *CG10407*. (*e*) *CG10863*. (*f–k*) RT-qPCR of the candidate genes in male reproductive tracts, including the seminal vesicles, of *JHRE^WT^-GFP* males with (MTP) or without (Ctrl) oral administration of methoprene. Each dot represents the levels of mRNA derived from 5 virgin males 7 days after eclosion. (*f*) RT-*JHRE-GFP* positive control. (*g*) *Kr-h1* positive control. (*h*) *Ldh*. (*i*) *Gdh*. (*j*) *CG10407*. (*k*) *CG10863*. (*l–n*) Immunostaining with anti-GFP antibody (green), phalloidin (magenta) and DAPI (Blue) of *Ldh-optGFP* virgin males 4 days after eclosion. (*l*) Image of the male reproductive tract. (*m*) Cross-section image of the seminal vesicle. (*n*) Magnified view of the seminal vesicle epithelial cells. (*o*) RT-qPCR of *Ldh* in the seminal vesicle of *Pde8-GAL4*-driven *Met* and *gce* RNAi flies. Control flies were obtained by crossing *w^1118^* with *Pde8-GAL4* driver. Each dot represents the levels of mRNA derived from 10 seminal vesicles of virgin males 7 days after eclosion. Values in *b–k* and *o* are presented as mean ± s.e.. Statistical analysis: Welch’s *t*‐test for *f–k*. Student’s *t*‐test for *o*. *p < 0.05, **p < 0.01, ***p < 0.001. n.s.: not significant.

In JH-responsive cells/tissues, JH signalling affects gene expression through Met and Gce [6,13]. Therefore, we searched for genes that are highly expressed in the seminal vesicles and potentially regulated by JH. First, we listed genes that might be highly expressed in the seminal vesicles using the results of proteomic analyses performed in two previous studies [30,31]. Among these proteome studies, one study used mixed samples of the seminal vesicles and sperm [30], while the other study only used sperm samples [31]. Comparing these two data sets, 66 proteins were considered candidates highly enriched in the seminal vesicles but not sperm (figure 4*a*, table 1). We also searched for the canonical Met/Gce binding E-box (CACGTG) or C-box (CACGCG) sequences in upstream regions of the 66 candidates. We found that many genes have E-boxes or C-boxes (electronic supplementary material, figure S2). Although the presence of these motifs does not directly imply that they are downstream genes of JH signalling, these data may provide clues to clarify the significance of JH signalling in seminal vesicles.

**Table 1.** Candidate proteins that are specifically and highly expressed in the seminal vesicles.

gene	avg_logFC (male reproductive gland)	*p*‐value	Gene	avg_logFC (male reproductive gland)	*p*‐value
CG10407	5.660312653	0	Prx2540-2	not found	not found
CG10863	3.085217476	8.75402 × 10^−11^	GstE12	not found	not found
Gdh	2.438033104	5.63537 × 10^−9^	Gdi	not found	not found
Ldh	2.38763833	2.66 × 10^−18^	Idgf3	not found	not found
Argk1	1.832550764	1.34915 × 10^−13^	Cam	not found	not found
regucalcin	1.310050249	1.75488 × 10^−14^	CG1648	not found	not found
GstD1	1.146826625	7.57 × 10^−18^	CG4520	not found	not found
Idgf4	0.629053414	0.000129652	CG7264	not found	not found
Vha26	0.506168485	0.021732058	CG14282	not found	not found
awd	−0.363285989	0.015698655	CG15125	not found	not found
Est-6	−0.43701005	1.56277 × 10^−5^	CG5177	not found	not found
Idh	−0.485521317	0.002354908	CG6287	not found	not found
Rack1	−0.502643883	0.000129842	Ogdh	not found	not found
Obp44a	not found	not found	Rpi	not found	not found
Gs1	not found	not found	CG34107	not found	not found
Dip-B	not found	not found	ND-19	not found	not found
scpr-C	not found	not found	ATPsynO	not found	not found
Inos	not found	not found	ND-B22	not found	not found
AdSS	not found	not found	RecQ5	not found	not found
Acsf2	not found	not found	Fkbp12	not found	not found
Bfc	not found	not found	Swim	not found	not found
CG11042	not found	not found	Mp20	not found	not found
Pglym78	not found	not found	Tm2	not found	not found
pyd3	not found	not found	Tm1	not found	not found
Pgk	not found	not found	Prm	not found	not found
Got2	not found	not found	Mf	not found	not found
LManII	not found	not found	Mhc	not found	not found
Fdh	not found	not found	Tsf1	not found	not found
CG8036	not found	not found	Zasp66	not found	not found
CG3609	not found	not found	porin	not found	not found
Mfe2	not found	not found	PPO1	not found	not found
Chd64	not found	not found	Sod2	not found	not found
Irp-1B	not found	not found	CG11815	not found	not found

avg_LogFC: average_Log fold change. ‘not found’ indicates that the presence of mRNA in the seminal vesicle cluster could not be confirmed on the Fly Cell Atlas.

**Table 2.** Candidate seminal vesicle-specific genes for suitable GAL4 identification.

	male reproductive gland		whole body	
gene	avg_logFC(5>)	*p*‐value (0.05<)	avg_logFC(5>)	*p*‐value (0.05<)
DIP-zeta	7.488148689	3.30132 × 10^−8^	5.467468262	6.72 × 10^−8^
CG14301	7.418711185	0	5.476506233	0
CG13460	7.363236427	5.49847 × 10^−6^	6.622333527	3.69572 × 10^−6^
CG9664	7.152559757	0	5.024883747	0
Obp93a	6.669476509	0.041431502	5.393202782	0.035683934
CG5612	6.657152176	0	6.221313	0
CG42828	6.422353268	0	6.050003529	0
CG18628	5.873726845	0	7.880100727	0
Pde8	5.734490395	0	5.000965118	0
NT5E-2	5.662868977	0	5.325617313	0
CG10407	5.660312653	0	5.038795471	0

avg_LogFC: average_Log fold change

Next, we browsed the *D. melanogaster* single-cell transcriptome database Fly Cell Atlas (https://flycellatlas.org/) [32] to obtain the gene expression dataset derived from the male reproductive glands. According to the Fly Cell Atlas dataset, the following four genes among the 66 candidate genes are highly enriched in the seminal vesicles as compared with other cells in the male reproductive glands (avg_logFC>2): *Ldh*, *Glutamate dehydrogenase* (*Gdh*), *CG10407* and *CG10863* (figure 4*a*, table 1). We then conducted reverse transcription-quantitative PCR (RT-qPCR) to confirm whether these genes were expressed in the seminal vesicles. The mRNA levels of all candidate genes were higher in the seminal vesicles compared with the testes and the male accessory glands (figure 4*b*–e).

To determine whether expression of these candidate genes is regulated by JH, we did RT-qPCR to measure mRNA levels in male reproductive tracts containing the seminal vesicles dissected from *JHRE^WT^-GFP* flies with and without methoprene administration. The mRNA levels of *JHRE-GFP* and the JH-responsive gene *Kr-h1*, used as positive controls, were upregulated by methoprene treatment (figure 4*f*,*g*). Among the candidate genes, *Ldh* mRNA levels were upregulated by methoprene treatment (figure 4*h*), while *Gdh*, *CG10407* and *CG10863* showed no change in mRNA levels (figure 4*i–k*). These results suggest that JH signalling in the seminal vesicle induces the expression of *Ldh*.

To confirm whether *Ldh* is expressed in the seminal vesicle epithelial cells, we used the transgenic strain, *Ldh-optGFP*, expressing *GFP*-tagged *Ldh* under the control of *Ldh* regulatory sequences [33,34]. We found that Ldh-optGFP signal was higher in the seminal vesicles, compared with other parts of male reproductive tracts (figure 4*l*). The magnified images show that *Ldh-optGFP* is expressed in the seminal vesicle epithelial cells (figure 4*m*,*n*), suggesting that *Ldh* is highly expressed in the seminal vesicle epithelial cells. Importantly, two canonical Met/Gce binding E-box sequences (CACGTG) are found in the *Ldh* locus, one motif is located in the *Ldh-RA* promoter region and the other motif is located within the first intron (electronic supplementary material, figure S3*a*). Then, we examined whether the expression of *Ldh* was regulated by Met and Gce. We found that *Ldh* mRNA level was decreased by a double knockdown of *Met* and *gce* in the seminal vesicle epithelial cells using *Pde8-GAL4* driver (figure 4*o*). Together, these results indicate that *Ldh* is a JH-responsive gene in the seminal vesicle epithelial cells. Finally, we conducted a luciferase-based assay in *D. melanogaster* S2 cultured cells using the promoter/enhancer region of *Ldh*. In cells expressing *luciferase* under the control of *JHRE*, methoprene treatment resulted in increased luciferase activity (electronic supplementary material, figure S3*b*), as reported previously [10]. However, in cells expressing luciferase driven by the region corresponding to −1160 + 1776 of *Ldh*, including the two E-boxes (electronic supplementary material, figure S3*a*), luciferase activity was not increased (electronic supplementary material, figure S3*b*). Together, these findings suggest that while *Ldh* expression in seminal vesicles is regulated by Met/Gce *in vivo*, *in vitro* JHA does not appear to influence *Ldh* expression by the two proximal E-boxes.

## 3. Discussion

The seminal vesicles are known to store, nourish and maintain sperm before they are transferred into the female reproductive tract [26]. In addition, the seminal vesicles act as secretory organs that may assist in producing seminal fluid proteins in some insects [35–40]. How seminal vesicles impart these functions or whether there are additional functions is not understood. In this study, we identified the seminal vesicle as a JH-responsive tissue in adult male *D. melanogaster*. While neither our current study nor previous studies have been able to clarify the biological significance of the action of JH on the seminal vesicles, our findings here implicate JH-dependent upregulation of the key metabolic enzyme, Ldh. Previous studies on the tasar silkmoth *Antheraea mylitta* has revealed that topical application of JH III to newly emerged adult males increases the concentration of total seminal vesicle proteins [41], suggesting the JH responsiveness of seminal vesicles might be evolutionarily conserved among insects.

How JH signalling in seminal vesicles supports male fertility or gamete quality is unknown. While JH signalling has been implicated in male fertility, JH is also involved in mating behaviour and memory in male *D. melanogaster* [24,25,42,43], making tissue-specific requirements for JH signalling challenging. We found that reducing JH signalling in the seminal vesicles did not reduce progeny (figure 3*e*). However, it should be noted that our male fertility measurements were done under non-competitive conditions. In the mosquito *A. aegypti*, loss of JH epoxidation in males does not affect the total number of eggs laid by wild-type females but does affect their reproductive fitness under competitive mating conditions [44]. It will be worthwhile to examine whether sperm competition is also affected by JH in *D. melanogaster*, and also whether it is affected by JH signalling in the seminal vesicles. Molecularly, how JH signalling affects seminal vesicle function remains unclear. JH is known to stimulate secretory activity in the male accessory glands of many insects [45]. In some insects, other than *D. melanogaster*, seminal vesicle epithelia contain secretory vesicles [37,38,40,46]. Together, these studies leave open the possibility that JH signalling affects the secretory activity of the seminal vesicles in *D. melanogaster*.

An important finding in this study is that the expression of *Ldh* in the seminal vesicles is upregulated by activation of JH signalling. While *Ldh* expression is known to be regulated by ecdysone signalling [47], our study is the first report that *Ldh* is also influenced by JH signalling. Whether JH induction of *Ldh* expression by JH signalling is central to seminal vesicle function is not yet clear. Neurobiological studies using *D. melanogaster* have shown that Ldh has an important role in supplying lactate from glial cells to neurons, known as a lactate shuttle, in response to neural activity in order to supply nutrients to neurons [48–50]. Considering the storage of many sperm in the seminal vesicles and the high expression of Ldh in the seminal vesicle epithelial cells, the lactate shuttle may exist between the sperm stored in the seminal vesicle and the seminal vesicle epithelial cells. It will be intriguing to examine whether JH signalling in the seminal vesicle changes in the quantity and/or quality of sperm.

An interesting previous study has reported that the seminal vesicle expresses multiple clock genes such as *period*, *Clock* (*Clk*) and *timeless*, all of which are necessary for generating proper circadian rhythm [51]. In the case of the mosquito *A. aegypti* female, it is reported that JH controls gene expression through a heterodimer of Met and circadian rhythm factor Cycle (CYC) [52]. It was also suggested that Met binds directly to CLK in *D. melanogaster* [53]. In addition, in the linden bug, *Pyrrhocoris apterus*, JH alters gene expression through Met, CLK and CYC in the gut [54]. Considering these previous reports and our results, circadian rhythm factors and JH may cooperate to regulate gene expression in the seminal vesicles.

In this study, we used both *JHRE-GFP* and *JHRR-LacZ* lines to analyse JH-responsive tissues. Unexpectedly, we found that *JHRR-LacZ* and *JHRE-GFP* differentially responded to methoprene administration in the seminal vesicle. JHRE-GFP signal was upregulated in response to methoprene feeding, while JHRR-LacZ signal was not. In addition, the expression pattern of these reporter lines was different in adult males. For example, JHRE-GFP signal was not observed in the male accessory gland, which has been reported as a JH-responsive tissue [24,55–57]. On the other hand, JHRR-LacZ signal was observed in the male accessory gland (electronic supplementary material, figure S1*b*). This difference may be due to the origin of *JHRE* and *JHRR. JHRE* in *JHRE^WT^-GFP* strain is derived from the *early trypsin* gene of *A. aegypti* [22,23], while *JHRR* is derived from *D. melanogaster Kr-h1* [14]. Alternatively, differences in reporter activity may reflect differences in response element number, with *JHRE-GFP* having eight tandem elements and *JHRR-LacZ* having one, or in genomic context, as both *JHRE^WT^-GFP* and *JHRE^Mut^-GFP* transgenes are inserted into the *attP2* site of the third chromosome while the *JHRR-LacZ* is randomly integrated into the third chromosome. Nonetheless, activities of both reporters are restricted to a limited number of cell types of male reproductive tracts. Previous studies reported that *Met-T2A-GAL4* and *gce-T2A-GAL4* labelled male accessory glands, ejaculatory duct, testes and seminal vesicles. On the other hand, we found that *JHRE-GFP* only labels cells in seminal vesicles and testes [21]. Considering that Met and Gce are expressed in almost all cell types of male reproductive tract [21], more comprehensive JH reporter strains will be needed in *D. melanogaster* and other insects in future studies.

Nevertheless, we propose that the *JHRE^WT^-GFP* and *JHRE^Mut^-GFP* strains [22] are nice tools to approximate JH signalling *in vivo* in adult male seminal vesicles. For example, in this study, we found that JHRE-GFP in the seminal vesicles is elevated after mating. This observation is consistent with the fact that JH titer is elevated after mating through the action of Ecdysis-triggering hormone [43]. Since *JHRE^WT^-GFP* strain has the tandem of eight JHREs [22], it may have the advantage of sensitivity for JH signalling. While direct measurements of actual JH titers are crucial [58], indirect approximation of JH titers through *JHRE^WT^-GFP* and *JHRE^Mut^-GFP* reporter activity in adult males is very easy and convenient. Use of JHRE-GFP signals in the seminal vesicles as a marker of JH signalling will facilitate future studies to increase our understanding of JH-dependent insect male physiology.

## 4. Materials and methods

### 4.1. *Drosophila melanogaster* strains and maintenance

*Drosophila melanogaster* flies were raised on a standard yeast-cornmeal-glucose fly medium (0.275 g agar, 5.0 g glucose, 4.5 g cornmeal, 2.0 g yeast extract, 150 μl propionic acid and 175 μl 10% butyl p-hydroxybenzoate (in 70% ethanol) in 50 ml water) at 25°C under a 12 : 12 h light/dark cycle. For the methoprene oral administration (figures 2*a,b,l,m* and 4*f*–*k**;* electronic supplementary material, figure S1*a*,*e*, *f*), virgin male flies were collected 0 to 8 h after eclosion, aged for 4 days on standard food, and then transferred for 3 days into new tubes in the presence of food supplemented with 60 µM methoprene (Sigma-Aldrich, St Louis, MO, PESTANAL 33375, racemic mixture; 1.5 M stock was prepared in ethanol) or 0.8% ethanol (control). To analyse the effect of mating (figure 3*a*–*d*), virgin male flies were collected at eclosion, aged for 4 days on standard food and then transferred for 2 days into new tubes in the presence of *w^1118^* 4 days after eclosion virgin females. The ratio of males to females in a vial for mating was 1 : 2. For experiments other than methoprene administration and mating, adult males were aged for 2 to 7 days on standard food.

The following transgenic strains were used: *how-GAL4* (Bloomington Drosophila stock center [BDSC] #1767), *JHAMT-GAL4* [59] (a gift from Sheng Li, South China Normal University, China), *JHRE^Mut^-GFP* [22], *JHRE^WT^-GFP* [22,23], *JHRR-LacZ* (a gift from Sheng Li), *KK control* (Vienna Drosophila resource center [VDRC] #60100), *Ldh-optGFP* (BDSC #94704), *Pde8-GAL4* (BDSC #65635), *UAS-GFP, mCD8::GFP* [60] (a gift from Kei Ito, University of Cologne, Germany), *UAS-gce-IR* (VDRC #101814), *UAS-jhamt-IR* (VDRC #103958), *UAS-Met-IR* (VDRC #45852), *UAS- mCD8::RFP* (BDSC #32219) and *UAS-stinger* (BDSC #84277).

### 4.2. Immunohistochemistry

The tissues were dissected in phosphate-buffered Saline (PBS) and fixed in 4% paraformaldehyde in PBS for 30–60 min at 25–27°C. The fixed samples were rinsed thrice in PBS, washed for 15 min with PBS containing 0.3% Triton X-100 (PBT), and treated with a blocking solution (2% bovine serum albumin in PBT; Sigma-Aldrich #A9647) for 1 h at 25–27°C or overnight at 4°C. The samples were incubated with a primary antibody in blocking solution overnight at 4°C. The primary antibodies used were as follows: chicken anti-GFP antibody (Abcam #ab13970, 1 : 2,000), mouse anti-LacZ (β-galactosidase; Developmental Studies Hybridoma Bank #40–1 a; 1 : 50), anti-RFP (Medical & Biological Laboratories PM005, 1 : 2,000). The samples were rinsed thrice with PBS and then washed for 15 min with PBT, followed by incubation with fluorophore (Alexa Fluor 488)-conjugated secondary antibodies (Thermo Fisher Scientific; 1 : 200) and in blocking solution for 2 h at RT or overnight at 4°C. Nuclear stains used in this study were 4',6-diamidino-2-phenylindole (DAPI; final concentration 1 μg ml^−1^ Sigma-Aldrich, St. Louis, MO, USA). F-Actin was stained with Alexa Fluor 568 phalloidin (1 : 200; Invitrogen, #A12380) or Alexa Fluor 647 phalloidin (1 : 500; Invitrogen, #A30107). For DAPI and phalloidin staining, after the incubation with the secondary antibodies, the samples were washed and then incubated with DAPI and phalloidin for at least 20 min at RT or overnight 4°C. After another round of washing, all the samples were mounted on glass slides using FluorSave reagent (Merck Millipore, #345789). For the quantification of JHRE-GFP signal (figures 2*a*–*f*, *l,m*, and 3*a*–c), only DAPI and phalloidin was stained after fixation. Confocal images were captured using the LSM 700 laser scanning confocal microscope (Carl Zeiss, Oberkochen, Germany). Quantification of immunostaining signal was conducted using the ImageJ software version 1.53q [61]. Fluorescence intensity of JHRE-GFP was normalized to the area of the seminal vesicle.

### 4.3. Sperm isolation

Sperm were isolated from 2 day old males carrying the JHRE-GFP reporter using the testes squash protocols from [62,63]. Briefly, testes were dissected in cold testes isolation buffer composed of 10 mM Tris-HCl pH 6.8, 183 mM KCl, 47 mM NaCl and 10 uM taxol (Thermo Fisher Scientific, #AC328420010) then placed on polysine adhesion slides (Thermo Fisher Scientific, #1254578). A coverslip was added on dissected testes, which were then squashed by dropping a pencil with the rubber eraser pointing down from a 0.5-inch height five times. Slides were placed in liquid nitrogen for approximately 7 s and then the coverslip was carefully removed with forceps. Testes and sperm were fixed in 4% paraformaldehyde for seven minutes, washed in 0.1% TritonX-100 in PBS (PBT), then washed twice with PBT containing 0.3% sodium deoxycholate (Thermo Fisher Scientific, #BP349-100) for 15 min each. Samples were washed twice more in PBT and then blocked with 2% BSA in PBT at 4°C overnight. Samples were stained with chicken anti-GFP (Aves Labs, #GFP-1020, 1 : 500) and mouse anti-1B1 (Developmental Studies Hybridoma Bank, 1 : 50) at 4°C for two nights. Samples were washed five times in PBT before incubating with donkey anti-mouse Cy3 (Jackson ImmunoResearch, #715-165-151, 1 : 500) and donkey anti-chicken 488 (Jackson ImmunoResearch, #703-545-155, 1 : 500) at 4°C for 4 h. Samples were washed in PBT, stained for DAPI at 1 : 10 000 (Thermo Fisher Scientific, #D1306, 1 : 10,000), and mounted in Vectashield (VectorLabs, #H-1000-10). Sperm were imaged using a Dragonfly 200 spinning disk confocal and images were processed using Imaris.

### 4.4. *Ex vivo* male reproductive tract culture

We collected *JHRE^WT^-GFP* virgin males 4 days after eclosion. The male reproductive tracts were dissected in Schneider’s Drosophila Medium (SDM; Thermo Fisher Scientific, #21720024), and male accessory glands were removed from the male reproductive tracts using forceps. Approximately 5–6 male reproductive tracts were immediately transferred to a dish containing 3 ml of SDM supplemented with 15% fetal calf serum and 0.6% penicillin-streptomycin with/without the addition of 1 µM methoprene (Sigma-Aldrich, St Louis, MO, PESTANAL 33375, racemic mixture; 1.5 M stock was prepared in ethanol) or 0.7% ethanol (control). The cultures were incubated at 25°C for 16 h, and the samples were immunostained to check the JHRE-GFP signal.

### 4.5. Screening of *GAL4* lines that label the seminal vesicle epithelial cells

To knock down *Met* and *gce* in the seminal vesicle, we needed a *GAL4* driver active in the seminal vesicle epithelial cells. For this purpose, we first surveyed which genes are highly and predominantly expressed in the seminal vesicles. Candidates of the seminal vesicle-specific genes were extracted from the single-cell transcriptome database, Fly Cell Atlas [32]. In the database, a transcriptomic cluster of the seminal vesicle was annotated in the 10 × Genomics dataset from the whole body and the male reproductive gland samples. We extracted the gene profile of the seminal vesicle cluster derived from the whole-body sample and the male reproductive gland sample. The two profiles of gene expression datasets were filtered by *p*‐value (*p*‐value < 0.05) and log fold change (avg_logFC>5). The avg_log FC indicates how specific the expression of a gene is in the certain cluster. Finally, 11 candidate genes were obtained (table 2). Of the published *GAL4* strains under the control of each of the 11 candidates, we promptly obtained *Pde8-GAL4* and confirmed the expression pattern of *Pde8-GAL4* in the seminal vesicle as described in the main text (figure 2*g*–j).

### 4.6. Quantifying progeny from male *Drosophila*

Virgin male flies were collected at eclosion, aged for 4 days on standard food and then transferred into new tubes in the presence of *w^1118^* 4 day old virgin females. The number of females was double that of the males. After 2 days, females were removed, and the males were kept alone for an additional 4 days. The males were then transferred to new tubes in the presence of *w^1118^* 4 day old virgin females. Here, males and females were paired one to one. After 1 day, the males were removed, and the females were kept in the same vial for another 6 days and then the number of pupae emerging from each vial was counted.

### 4.7. Screening of candidate genes that are specifically and highly expressed in the seminal vesicles

Candidate proteins highly enriched in the seminal vesicle were determined by comparing the two independent proteomics datasets. One dataset [30] annotates 168 proteins as being enriched in the seminal vesicle and/or sperm stored in the seminal vesicle. Another dataset [31] annotates 381 proteins as being enriched in the sperm isolated from the seminal vesicle. We found that two datasets share 102 proteins, suggesting that these shared proteins are enriched in the sperm but not the seminal vesicle, with the remaining 66 proteins (168 minus 102) as candidate proteins enriched in the seminal vesicle (table 1). Next, we checked whether each of the genes encoding the 66 proteins is predominantly expressed in the seminal vesicles by the single-cell transcriptome database Fly Cell Atlas [32]. We extracted gene profiles of the seminal vesicle cluster in male reproductive gland sample. The candidate genes were filtered by *p*‐value (*p* < 0.05) and log fold change (avg_logFC>5). Finally, we obtained four candidate genes, *Ldh*, *Gdh*, *CG10407* and *CG10863*.

### 4.8. Reverse transcription-quantitative PCR (RT-qPCR)

RNA from tissues was extracted using RNAiso Plus (Takara Bio) and reverse-transcribed using ReverTra Ace qPCR RT Master Mix with gDNA Remover (TOYOBO). Synthesized cDNA samples were used as templates for quantitative PCR using THUNDERBIRD SYBR qPCR Mix (TOYOBO) on a Thermal Cycler Dice Real Time System (Takara Bio). The amount of target RNA was normalized to the endogenous control *ribosomal protein 49* gene (*rp49*) and the relative fold change was calculated. The expression levels of each gene were compared using the ΔΔCt method [64]. The following primers were used for this analysis: *rp49* F (5′-CGGATCGATATGCTAAGCTGT-3′), *rp49* R (5′-GCGCTTGTTCGATCCGTA-3′), *GFP* F (5′-GAACCGCATCGAGCTGAA-3′), *GFP* R (5′-TGCTTGTCGGCCATGATATAG-3′), *CG10407* F (5′-ACTGGACAACAGCCAAACCTC-3′), *CG10407* R (5′-GTGTCTAGGTCGGGTGCATTG-3′), *Ldh* F (5′-CGTTTGGTCTGGAGTGAACA-3′), *Ldh* R (5′-GCAGCTCGTTCCACTTCTCT-3′), *Gdh* F (5′-GGAGGACTACAAGAACGAGCA-3′), *Gdh* R (5′-CAGCCACTCGAAGAAGGAGA-3′), *CG10863* F (5′-CATCGGACTGGGCACCTATAC-3′), *CG10863* R (5′-TTCTCGTAGAAATAGGCGGTGTC-3′), *Kr-h1* F (5′-TCACACATCAAGAAGCCAACT-3′) and *Kr-h1* R (5′-GCTGGTTGGCGGAATAGTAA-3′).

### 4.9. Construction of luciferase reporter plasmids

We amplified a −1160 to+1776 bp upstream region of *Ldh* from *w^1118^* genomic DNA using primers (Fwd: 5′-ACTGAGCTCTACAGATCTCTTGAGGACTCTCTATGG-3′, Rev: 5′-TGACTCGAGTAACTTTAATATTCCGCCAAAGAAAGC-3′) to add *Sac1* and *Xho1* sites to the 5′ and 3′ ends, respectively. These amplified *Ldh* upstream regions were digested with *Sac1* and *Xho1* and ligated into a *Sac1-Xho1*-digested pGL3-Basic vector luciferase reporter plasmid (Promega #E175A).

### 4.10. Transfection and luciferase reporter assays

S2 cells were seeded in 500 µl Schneider’s Drosophila Medium (SDM; Thermo Fisher Scientific, #21720024) supplemented with 10% fetal bovine serum (FBS; Thermo Fisher Scientific, #10270106) and 1% penicillin-streptomycin solution (Fujifilm Wako, #168-23191) in a 24-well plate (TPP, #92424) 24 h before transfection. Transfection of S2 cells was performed using the Effectene Transfection Reagent (Qiagen, #301425). *JHRE^WT^-luc* and *JHRE^Mut^-luc* plasmids (generous gifts from Dr Marek Jindra) [10], *Ldh-luc* plasmid (in this study) and an empty pGL3-Basic plasmid were transfected along with the luciferase reporter plasmids. The *Copia Renilla* control plasmid (Addgene, #38093) was used as the reference. The cells were incubated for 48 hr after transfection. Subsequently, 5 µl of 99.5% EtOH (Nacalai Tesque, #14712-63) or 100 µM methoprene (Fujifilm #136-17621) in 99.5% EtOH was added and incubated for 8 h. Then, they were processed by using the Dual-Luciferase Reporter Assay System (Promega, #E1960) in accordance with the manufacturer’s instructions and were analysed with Fluoroskan ascent FL (Thermo Fisher Scientific).

## 5. Statistical analysis

All experiments were performed independently at least twice. The sample sizes were chosen based on the number of independent experiments required for statistical significance and technical feasibility. The experiments were not randomized, and the investigators were not blinded. All statistical analyses were performed using the ‘R’ software version 4.0.3. Details of the statistical analyses are described in figure legends.

## Data Availability

Electronic supplementary material 2 provides raw numerical data generated in this study. All other source data are provided upon request to R.N. Supplementary material is available online [65].

## References

[1] Li K, Jia QQ, Li S. 2019 Juvenile hormone signaling—a mini review. Insect Sci. **26**, 600–606. (10.1111/1744-7917.12614) 29888456

[2] Noriega FG. 2014 Juvenile hormone biosynthesis in insects: what is new, what do we know, and what questions remain? Int. Sch. Res. Notices **2014**, 967361. (10.1155/2014/967361) 27382622 PMC4897325

[3] Qu Z, Bendena WG, Tobe SS, Hui JHL. 2018 Juvenile hormone and sesquiterpenoids in arthropods: biosynthesis, signaling, and role of MicroRNA. J. Steroid Biochem. Mol. Biol. **184**, 69–76. (10.1016/j.jsbmb.2018.01.013) 29355708

[4] Riddiford LM. 2020 Rhodnius, golden oil, and met: a history of juvenile hormone research. Front. Cell Dev. Biol. **8**, 679. (10.3389/fcell.2020.00679) 32850806 PMC7426621

[5] Goodman WG, Cusson M. 2012 The juvenile hormones. In Insect endocrinology (ed. LI Gilbert), pp. 310–365. London, UK: Academic Press. (10.1016/B978-0-12-384749-2.10008-1)

[6] Rivera-Pérez C, Clifton ME, Noriega FG, Jindra M. 2020 Juvenile hormone regulation and action. In Advances in invertebrate (neuro)endocrinology, pp. 1–76. Palm Bay, FL: Apple Academic Press. (10.1201/9781003029861-1)

[7] Shinoda T. 2021 Juvenile hormone. In Handbook of hormones: comparative endocrinology for basic and clinical research (eds H Ando, K Ukena, S Nagata), pp. 987–989. London, UK: Academic Press.

[8] Kurogi Y, Mizuno Y, Imura E, Niwa R. 2021 Neuroendocrine regulation of reproductive dormancy in the fruit fly Drosophila melanogaster: a review of juvenile hormone-dependent regulation. Front. Ecol. Evol. **9**, 715029. (10.3389/fevo.2021.715029)

[9] Abdou MA *et al*. 2011 Drosophila met and gce are partially redundant in transducing juvenile hormone action. Insect Biochem. Mol. Biol. **41**, 938–945. (10.1016/j.ibmb.2011.09.003) 21968404

[10] Jindra M, Uhlirova M, Charles JP, Smykal V, Hill RJ. 2015 Genetic evidence for function of the bHLH-PAS protein Gce/Met as a juvenile hormone receptor. PLoS Genet. **11**, e1005394. (10.1371/journal.pgen.1005394) 26161662 PMC4498814

[11] Wilson TG. 1996 Genetic evidence that mutants of the methoprene-tolerant gene of Drosophila melanogaster are null mutants. Arch. Insect Biochem. Physiol. **32**, 641–649. (10.1002/(SICI)1520-6327(1996)32:3/4<641::AID-ARCH35>3.0.CO;2-A) 8756311

[12] Tumova S, Dolezel D, Jindra M. 2024 Conserved and unique roles of bHLH-PAS transcription factors in insects—from clock to hormone reception. J. Mol. Biol. **436**, 168332. (10.1016/j.jmb.2023.168332) 39491146

[13] Jindra M, Bellés X, Shinoda T. 2015 Molecular basis of juvenile hormone signaling. Curr. Opin. Insect Sci. **11**, 39–46. (10.1016/j.cois.2015.08.004) 28285758

[14] He Q, Wen D, Jia Q, Cui C, Wang J, Palli SR, Li S. 2014 Heat shock protein 83 (Hsp83) facilitates methoprene-tolerant (Met) nuclear import to modulate juvenile hormone signaling. J. Biol. Chem. **289**, 27874–27885. (10.1074/jbc.M114.582825) 25122763 PMC4183821

[15] Kayukawa T *et al*. 2012 Transcriptional regulation of juvenile hormone-mediated induction of Krüppel homolog 1, a repressor of insect metamorphosis. Proc. Natl Acad. Sci. USA **109**, 11729–11734. (10.1073/pnas.1204951109) 22753472 PMC3406821

[16] Li M, Mead EA, Zhu J. 2011 Heterodimer of two bHLH-PAS proteins mediates juvenile hormone-induced gene expression. Proc. Natl Acad. Sci. USA **108**, 638–643. (10.1073/pnas.1013914108) 21187375 PMC3021087

[17] Li M, Liu P, Wiley JD, Ojani R, Bevan DR, Li J, Zhu J. 2014 A steroid receptor coactivator acts as the DNA-binding partner of the methoprene-tolerant protein in regulating juvenile hormone response genes. Mol. Cell. Endocrinol. **394**, 47–58. (10.1016/j.mce.2014.06.021) 25004255 PMC4163509

[18] Zhang Z, Xu J, Sheng Z, Sui Y, Palli SR. 2011 Steroid receptor co-activator is required for juvenile hormone signal transduction through a bHLH-PAS transcription factor, methoprene tolerant. J. Biol. Chem. **286**, 8437–8447. (10.1074/jbc.M110.191684) 21190938 PMC3048728

[19] Zhang X, Li S, Liu S. 2021 Juvenile hormone studies in Drosophila melanogaster. Front. Physiol. **12**, 785320. (10.3389/fphys.2021.785320) 35222061 PMC8867211

[20] Riddiford LM, Truman JW, Mirth CK, Shen YC. 2010 A role for juvenile hormone in the prepupal development of Drosophila melanogaster. Development **137**, 1117–1126. (10.1242/dev.037218) 20181742 PMC2835327

[21] Baumann AA, Texada MJ, Chen HM, Etheredge JN, Miller DL, Picard S, Warner R, Truman JW, Riddiford LM. 2017 Genetic tools to study juvenile hormone action in Drosophila. Sci. Rep. **7**, 2132. (10.1038/s41598-017-02264-4) 28522854 PMC5437021

[22] Barton LJ, Sanny J, Packard Dawson E, Nouzova M, Noriega FG, Stadtfeld M, Lehmann R. 2024 Juvenile hormones direct primordial germ cell migration to the embryonic gonad. Curr. Biol. **34**, 505–518.(10.1016/j.cub.2023.12.033) 38215744 PMC10872347

[23] Noriega FG, Shah DK, Wells MA. 1997 Juvenile hormone controls early trypsin gene transcription in the midgut of Aedes aegypti. Insect Mol. Biol. **6**, 63–66. (10.1046/j.1365-2583.1997.00154.x) 9013256

[24] Wolfner MF, Partridge L, Lewin S, Kalb JM, Chapman T, Herndon LA. 1997 Mating and hormonal triggers regulate accessory gland gene expression in male Drosophila. J. Insect Physiol. **43**, 1117–1123. (10.1016/s0022-1910(97)00062-0) 12770484

[25] Meiselman MR, Ganguly A, Dahanukar A, Adams ME. 2022 Endocrine modulation of primary chemosensory neurons regulates Drosophila courtship behavior. PLoS Genet. **18**, e1010357. (10.1371/journal.pgen.1010357) 35998183 PMC9439213

[26] Chapman RF. 2012 The insects structure and function, 5th edn. Cambridge, UK: Cambridge University Press.

[27] Fyrberg C, Becker J, Barthmaier P, Mahaffey J, Fyrberg E. 1997 A Drosophila muscle-specific gene related to the mouse quaking locus. Gene **197**, 315–323. (10.1016/s0378-1119(97)00278-3) 9332381

[28] Shinoda T, Itoyama K. 2003 Juvenile hormone acid methyltransferase: a key regulatory enzyme for insect metamorphosis. Proc. Natl Acad. Sci. USA **100**, 11986–11991. (10.1073/pnas.2134232100) 14530389 PMC218700

[29] Niwa R, Niimi T, Honda N, Yoshiyama M, Itoyama K, Kataoka H, Shinoda T. 2008 Juvenile hormone acid O-methyltransferase in Drosophila melanogaster. Insect Biochem. Mol. Biol. **38**, 714–720. (10.1016/j.ibmb.2008.04.003) 18549957

[30] Takemori N, Yamamoto MT. 2009 Proteome mapping of the Drosophila melanogaster male reproductive system. Proteomics **9**, 2484–2493. (10.1002/pmic.200800795) 19343724

[31] Dorus S, Busby SA, Gerike U, Shabanowitz J, Hunt DF, Karr TL. 2006 Genomic and functional evolution of the Drosophila melanogaster sperm proteome. Nat. Genet. **38**, 1440–1445. (10.1038/ng1915) 17099714

[32] Li H *et al*. 2022 Fly cell Atlas: a single-nucleus transcriptomic atlas of the adult fruit fly. Science **375**, eabk2432. (10.1126/science.abk2432) 35239393 PMC8944923

[33] Bawa S *et al*. 2020 Drosophila TRIM32 cooperates with glycolytic enzymes to promote cell growth. eLife **9**, e52358. (10.7554/eLife.52358) 32223900 PMC7105379

[34] Li H *et al*. 2017 Drosophila larvae synthesize the putative oncometabolite L-2-hydroxyglutarate during normal developmental growth . Proc. Natl Acad. Sci. USA **114**, 1353–1358. (10.1073/pnas.1614102114) 28115720 PMC5307464

[35] Riemann JG, Thorson BJ. 1976 Ultrastructure of the vasa deferentia of the mediterranean flour moth. J. Morphol. **149**, 483–505. (10.1002/jmor.1051490404) 30301288

[36] Couche GA, Gillott C. 1988 Development of secretory activity in the seminal vesicle of the male migratory grasshopper, Melanoplus sanguinipes (fabr.) (Orthoptera: Acrididae). Int. J. Insect Morphol. Embryol. **17**, 51–61. (10.1016/0020-7322(88)90030-X)

[37] Xie S, Hua B. 2010 Ultrastructure of the seminal vesicle and sperm storage in Panorpidae (Insecta: Mecoptera). Micron **41**, 760–768. (10.1016/j.micron.2010.05.012) 20646927

[38] Viscuso R, Brundo MV, Marletta A, Vitale DGM. 2015 Fine structure of male genital tracts of some Acrididae and Tettigoniidae (Insecta: Orthoptera). Acta Zool. **96**, 418–427. (10.1111/azo.12084)

[39] Spiegel CN, Bretas JAC, Peixoto AA, Vigoder FM, Bruno RV, Soares MJ. 2013 Fine structure of the male reproductive system and reproductive behavior of Lutzomyia longipalpis sandflies (Diptera: Psychodidae: Phlebotominae). PLoS One **8**, e74898. (10.1371/journal.pone.0074898) 24058637 PMC3772895

[40] Lyu QH, Zhang BB, Hua BZ. 2018 Ultrastructure and function of the seminal vesicle of Bittacidae (Insecta: Mecoptera). Arthropod Struct. Dev. **47**, 173–179. (10.1016/j.asd.2018.02.001) 29425772

[41] Pendam VR, Tembhare DB. 2013 Effect of JH III and β-ecdysone on seminal vesicle protein secretion in the tropical tasar silkmoth, Antheraea mylitta (Drury) (Lepidoptera: Saturniidae). Int. J. Wild Silkmoth Silk **17**, 43–48. (10.51011/ijwss.17.0_43)

[42] Wijesekera TP, Saurabh S, Dauwalder B. 2016 Juvenile hormone is required in adult males for Drosophila courtship. PLoS One **11**, e0151912. (10.1371/journal.pone.0151912) 27003411 PMC4803231

[43] Lee SS, Ding Y, Karapetians N, Rivera-Perez C, Noriega FG, Adams ME. 2017 Hormonal signaling cascade during an early-adult critical period required for courtship memory retention in Drosophila. Curr. Biol. **27**, 2798–2809.(10.1016/j.cub.2017.08.017) 28918947

[44] Nouzova M *et al*. 2021 Epoxidation of juvenile hormone was a key innovation improving insect reproductive fitness. Proc. Natl Acad. Sci. USA **118**, e2109381118. (10.1073/pnas.2109381118) 34697248 PMC8609300

[45] Raikhel AS, Brown MR, Belles X. 2005 Hormonal control of reproductive processes. In Comprehensive molecular insect science (ed. LI Gilbert), pp. 433–491. Amsterdam, The Netherlands: Elsevier. (10.1016/B0-44-451924-6/00040-5)

[46] Fausto AM, Gambellini G, Taddei AR, Maroli M, Mazzini M. 2000 Ultrastructure of the seminal vesicle of Phlebotomus perniciosus Newstead (Diptera, Psychodidae). Tissue Cell **32**, 228–237. (10.1054/tice.2000.0110) 11037793

[47] Abu-Shumays RL, Fristrom JW. 1997 IMP-L3, a 20-hydroxyecdysone-responsive gene encodes Drosophila lactate dehydrogenase: structural characterization and developmental studies. Dev. Genet. **20**, 11–22. (10.1002/(SICI)1520-6408(1997)20:1<11::AID-DVG2>3.0.CO;2-C) 9094207

[48] Volkenhoff A, Weiler A, Letzel M, Stehling M, Klämbt C, Schirmeier S. 2015 Glial glycolysis is essential for neuronal survival in Drosophila. Cell Metab. **22**, 437–447. (10.1016/j.cmet.2015.07.006) 26235423

[49] Liu L, MacKenzie KR, Putluri N, Maletić-Savatić M, Bellen HJ. 2017 The glia-neuron lactate shuttle and elevated ROS promote lipid synthesis in neurons and lipid droplet accumulation in glia via APOE/D. Cell Metab. **26**, 719–737.(10.1016/j.cmet.2017.08.024) 28965825 PMC5677551

[50] Brooks GA. 2018 The science and translation of lactate shuttle theory. Cell Metab. **27**, 757–785. (10.1016/j.cmet.2018.03.008) 29617642

[51] Beaver LM, Gvakharia BO, Vollintine TS, Hege DM, Stanewsky R, Giebultowicz JM. 2002 Loss of circadian clock function decreases reproductive fitness in males of Drosophila melanogaster. Proc. Natl Acad. Sci. USA **99**, 2134–2139. (10.1073/pnas.032426699) 11854509 PMC122331

[52] Shin SW, Zou Z, Saha TT, Raikhel AS. 2012 BHLH-PAS heterodimer of methoprene-tolerant and cycle mediates circadian expression of juvenile hormone-induced mosquito genes. Proc. Natl Acad. Sci. USA **109**, 16576–16581. (10.1073/pnas.1214209109) 23012454 PMC3478602

[53] He L, Wu B, Shi J, Du J, Zhao Z. 2023 Regulation of feeding and energy homeostasis by clock-mediated gart in Drosophila. Cell Rep. **42**, 112912. (10.1016/j.celrep.2023.112912) 37531254

[54] Bajgar A, Jindra M, Dolezel D. 2013 Autonomous regulation of the insect gut by circadian genes acting downstream of juvenile hormone signaling. Proc. Natl Acad. Sci. USA **110**, 4416–4421. (10.1073/pnas.1217060110) 23442387 PMC3600444

[55] Shemshedini L, Lanoue M, Wilson TG. 1990 Evidence for a juvenile hormone receptor involved in protein synthesis in Drosophila melanogaster. J. Biol. Chem. **265**, 1913–1918. (10.1016/S0021-9258(19)39917-X) 2105312

[56] Wilson TG, DeMoor S, Lei J. 2003 Juvenile hormone involvement in Drosophila melanogaster male reproduction as suggested by the Methoprene-tolerant^27^ mutant phenotype. Insect Biochem. Mol. Biol. **33**, 1167–1175. (10.1016/j.ibmb.2003.06.007) 14599489

[57] Yamamoto K, Chadarevian A, Pellegrini M. 1988 Juvenile hormone action mediated in male accessory glands of Drosophila by calcium and kinase C. Science **239**, 916–919. (10.1126/science.3124270) 3124270

[58] Rivera-Perez C, Nouzova M, Noriega FG. 2012 A quantitative assay for the juvenile hormones and their precursors using fluorescent tags. PLoS One **7**, e43784. (10.1371/journal.pone.0043784) 22928033 PMC3425502

[59] Wen D *et al*. 2015 Methyl farnesoate plays a dual role in regulating Drosophila metamorphosis. PLoS Genet. **11**, e1005038. (10.1371/journal.pgen.1005038) 25774983 PMC4361637

[60] Ito K, Suzuki K, Estes P, Ramaswami M, Yamamoto D, Strausfeld NJ. 1998 The organization of extrinsic neurons and their implications in the functional roles of the mushroom bodies in Drosophila melanogaster meigen. Learn. Mem. **5**, 52–77. 10454372 PMC311240

[61] Schneider CA, Rasband WS, Eliceiri KW. 2012 NIH image to ImageJ: 25 years of image analysis. Nat. Methods. **9**, 671–675. (10.1038/nmeth.2089) 22930834 PMC5554542

[62] Casal J, Gonzalez C, Wandosell F, Avila J, Ripoll P. 1990 Abnormal meiotic spindles cause a cascade of defects during spermatogenesis in asp males of Drosophila. Development **108**, 251–260. (10.1242/dev.108.2.251) 2112454

[63] Shao L *et al*. 2023 Eukaryotic translation initiation factor eIF4E-5 is required for spermiogenesis in Drosophila melanogaster. Development **150**, dev200477. (10.1242/dev.200477) 36695474

[64] Livak KJ, Schmittgen TD. 2001 Analysis of relative gene expression data using real-time quantitative PCR and the 2^-ΔΔC(T)^ method. Methods **25**, 402–408. (10.1006/meth.2001.1262) 11846609

[65] Kurogi Y, Mizuno Y, Hayashi R, Goyins K, Okamoto N, Barton L *et al*. 2024 Supplementary material from The seminal vesicle is a juvenile hormone-responsive tissue in adult male Drosophila melanogaster. Figshare (10.6084/m9.figshare.c.7576624)39689858

